# Supplementary material for: An Isolated Arthrobacter sp. Enhances Rice (Oryza sativa L.) Plant Growth
Source: Microorganisms. 2022 Jun 9;10(6):1187. doi: 10.3390/microorganisms10061187 (PMC9228311; doi:10.3390/microorganisms10061187)
Supplement: Supplementary file 1 [file microorganisms-10-01187-s001.zip › microorganisms-1760479-supplementary.pdf]

An Isolated *Arthrobacter* sp. Enhances Rice (*Oryza Sativa* L.) Plant Growth

Geeta Chhetri†, Inhyup Kim†, Minchung Kang, Yoonseop So, Jiyoun Kim and Taegun Seo*

Department of Life Science, Dongguk University-Seoul, Goyang 10326, South Korea

*Correspondence: tseo@dongguk.edu; Tel.: +82-31-961-5135

†These authors contributed equally to this work.

***Corresponding author:**

Taegun Seo

Department of Life Science, Dongguk University-Seoul, Goyang, 10326, South Korea

Tel: +82-31-961-5135

Fax: +82-31-961-5348

E-mail: tseo@dongguk.edu

Table S1. Presence of genes associated with plant growth promoting in genomes of strain GN70.

Accession number	Start	Stop	Locus tag	Protein Name
**Indole acetic acid**				
NZ_JAHHIO010000002.1	198626	199444	KKI43_RS03285	MULTISPECIES: indole-3-glycerol phosphate synthase (***trpC)***
NZ_JAHHIO010000010.1	140595	141686	KKI43_RS12540	MULTISPECIES: tryptophan--tRNA ligase (***trpS*)**
NZ_JAHHIO010000004.1	237142	237996	KKI43_RS06875	MULTISPECIES: tryptophan 2,3-dioxygenase (***kynA)***
NZ_JAHHIO010000002.1	199556	200968	KKI43_RS03290	MULTISPECIES: tryptophan synthase subunit beta (***trpB)***
NZ_JAHHIO010000002.1	200968	201792	KKI43_RS03295	MULTISPECIES: tryptophan synthase subunit alpha (***trpA)***
**Siderophore**				
NZ_JAHHIO010000003.1	241307	242146	KKI43_RS05390	MULTISPECIES: siderophore-interacting protein
NZ_JAHHIO010000034.1	8017	9045	KKI43_RS23700	MULTISPECIES: siderophore-interacting protein
NZ_JAHHIO010000034.1	12070	13137	KKI43_RS23720	MULTISPECIES: iron-siderophore ABC transporter substrate-binding protein
**Nitrogen fixation**				
NZ_JAHHIO010000010.1	12870	13352	KKI43_RS12000	MULTISPECIES: SUF system NifU family Fe-S cluster assembly protein (***nifU)***
**Acetolactate synthase production**				
NZ_JAHHIO010000012.1	94992	96017	KKI43_RS13890	MULTISPECIES: ketol-acid reductoisomerase ***ilvC***
NZ_JAHHIO010000012.1	98886	100607	KKI43_RS13905	MULTISPECIES: dihydroxy-acid dehydratase ***ilvD***
NZ_JAHHIO010000015.1	92660	94540	KKI43_RS15885	MULTISPECIES: dihydroxy-acid dehydratase ***ilvD***
NZ_JAHHIO010000012.1	96110	96622	KKI43_RS13895	MULTISPECIES: acetolactate synthase small subunit (***ilvN*)**
**Phosphate solubilization**				
NZ_JAHHIO010000013.1	19550	20485	KKI43_RS14235	MULTISPECIES: phosphate/phosphite/phosphonate ABC transporter substrate- binding protein
NZ_JAHHIO010000013.1	21246	22067	KKI43_RS14245	MULTISPECIES: phosphonate ABC transporter, permease protein (***phnE*)**
**Extracellular polysaccharide**				
NZ_JAHHIO010000013.1	149801	150763	KKI43_RS14820	MULTISPECIES: polysaccharide deacetylase
NZ_JAHHIO010000021.1	16256	17494	KKI43_RS19115	MULTISPECIES: polysaccharide biosynthesis protein
NZ_JAHHIO010000001.1	420280	421185	KKI43_RS02035	MULTISPECIES: polysaccharide deacetylase family protein

NZ_JAHHIO010000001.1	445679	447181	KKI43_RS02155	MULTISPECIES: polysaccharide biosynthesis tyrosine autokinase
NZ_JAHHIO010000010.1	20535	21941	KKI43_RS12025	MULTISPECIES: polysaccharide deacetylase family protein
NZ_JAHHIO010000014.1	14744	15757	KKI43_RS14905	MULTISPECIES: polysaccharide deacetylase family protein
NZ_JAHHIO010000014.1	17192	19054	KKI43_RS14920	MULTISPECIES: polysaccharide deacetylase family protein
NZ_JAHHIO010000015.1	98489	99418	KKI43_RS15910	MULTISPECIES: polysaccharide deacetylase family protein
NZ_JAHHIO010000015.1	102102	103031	KKI43_RS15935	MULTISPECIES: polysaccharide deacetylase family protein
NZ_JAHHIO010000015.1	116353	117675	KKI43_RS16005	MULTISPECIES: polysaccharide deacetylase family protein
NZ_JAHHIO010000021.1	26541	28982	KKI43_RS19150	MULTISPECIES: polysaccharide deacetylase family protein
NZ_JAHHIO010000003.1	86998	87885	KKI43_RS04615	MULTISPECIES: polysaccharide deacetylase family protein
NZ_JAHHIO010000005.1	192046	192960	KKI43_RS08125	MULTISPECIES: polysaccharide deacetylase family protein
NZ_JAHHIO010000019.1	110687	113383	KKI43_RS18510	MULTISPECIES: polysaccharide lyase 8 family protein

Table S2. NCBI annotation revealed the different genes involve in cold-shock, heat-shock, nitrogen regulatory protein, antioxidant response, riboflavin synthesis and phenazine biosynthesis in the genome of strain GN70.

Cold shock proteins					
**Accession number**	**Start**	**Stop**	**Locus tag**	**Length**	**Protein Name**
NZ_JAHHIO010000013.1	59680	59883	KKI43_RS14430	67	MULTISPECIES: cold-shock protein
NZ_JAHHIO010000015.1	55628	55831	KKI43_RS15690	67	MULTISPECIES: cold-shock protein
NZ_JAHHIO010000018.1	45620	45823	KKI43_RS17585	67	MULTISPECIES: cold-shock protein
NZ_JAHHIO010000022.1	23322	23525	KKI43_RS19670	67	MULTISPECIES: cold-shock protein
NZ_JAHHIO010000003.1	244174	244377	KKI43_RS05410	67	MULTISPECIES: cold-shock protein
NZ_JAHHIO010000032.1	6564	6767	KKI43_RS23265	67	MULTISPECIES: cold-shock protein
NZ_JAHHIO010000009.1	147499	147702	KKI43_RS11835	67	MULTISPECIES: cold-shock protein
NZ_JAHHIO010000003.1	258234	258617	KKI43_RS05475	127	MULTISPECIES: cold shock domain-containing protein
**Heat shock proteins**					
NZ_JAHHIO010000019.1	53504	54517	KKI43_RS18250	337	MULTISPECIES: heat-inducible transcriptional repressor (***HrcA***)
NZ_JAHHIO010000019.1	52314	53444	KKI43_RS18245	376	MULTISPECIES: molecular chaperone (***DnaJ***)
NZ_JAHHIO010000016.1	128016	128987	KKI43_RS16690	323	MULTISPECIES: domain-containing protein ***(DnaJ)***
NZ_JAHHIO010000029.1	52438	52971	KKI43_RS22715	177	MULTISPECIES: domain-containing protein ***(DnaJ)***
NZ_JAHHIO010000016.1	125213	127075	KKI43_RS16680	620	MULTISPECIES: molecular chaperone (***DnaK***)
NZ_JAHHIO010000001.1	127378	128988	KKI43_RS00640	536	MULTISPECIES: chaperonin (***GroEL***)
NZ_JAHHIO010000003.1	246159	247787	KKI43_RS05420	542	MULTISPECIES: chaperonin (***GroE*L**)
NZ_JAHHIO010000001.1	129075	129371	KKI43_RS00645	98	MULTISPECIES: co-chaperone (***GroES***)
NZ_JAHHIO010000001.1	307873	308742	KKI43_RS01535	289	MULTISPECIES: zinc metalloprotease (***HtpX***)
NZ_JAHHIO010000016.1	127078	127749	KKI43_RS16685	223	MULTISPECIES: nucleotide exchange factor (***GrpE***)
**Nitrogen regulatory proteins**					

NZ_JAHHIO010000001.1	98097	98954	KKI43_RS00495	285	MULTISPECIES: carbon-nitrogen hydrolase
NZ_JAHHIO010000012.1	49508	49846	KKI43_RS13680	112	MULTISPECIES: P-II family nitrogen regulator
NZ_JAHHIO010000013.1	142235	142576	KKI43_RS14780	113	MULTISPECIES: P-II family nitrogen regulator
NZ_JAHHIO010000027.1	52363	53169	KKI43_RS22135	268	MULTISPECIES: carbon-nitrogen hydrolase family protein
**Antioxidant response proteins**					
NZ_JAHHIO010000018.1	93735	94232	KKI43_RS17830	165	MULTISPECIES: glutathione peroxidase
NZ_JAHHIO010000001.1	306390	307793	KKI43_RS01530	467	MULTISPECIES: deferrochelatase/peroxidase (***EfeB***)
NZ_JAHHIO010000019.1	89281	91512	KKI43_RS18425	743	MULTISPECIES: catalase/peroxidase HPI (***katG***)
NZ_JAHHIO010000012.1	103213	103686	KKI43_RS13930	157	MULTISPECIES: thioredoxin-dependent thiol peroxidase (***bcp***)
NZ_JAHHIO010000007.1	73325	73792	KKI43_RS09555	155	MULTISPECIES: thioredoxin-dependent thiol peroxidase (***bcp***)
NZ_JAHHIO010000001.1	310769	312220	KKI43_RS01550	483	MULTISPECIES: catalase
NZ_JAHHIO010000019.1	89281	91512	KKI43_RS18425	743	MULTISPECIES: catalase/peroxidase HPI (***katG***)
NZ_JAHHIO010000005.1	220346	220969	KKI43_RS08230	207	MULTISPECIES: superoxide dismutase
NZ_JAHHIO010000020.1	19039	19740	KKI43_RS18665	233	MULTISPECIES: chlorite dismutase family protein
**Riboflavin synthase**					
NZ_JAHHIO010000002.1	189910	190548	KKI43_RS03226	212	MULTISPECIES: riboflavin synthase
**Phenazine biosynthesis**					
NZ_JAHHIO010000019.1	85797	86048	KKI43_RS18405	83	MULTISPECIES: PhzF family phenazine biosynthesis protein
NZ_JAHHIO010000008.1	172274	173125	KKI43_RS10955	283	MULTISPECIES: PhzF family phenazine biosynthesis protein
